# PolyHIPE Derived Freestanding 3D Carbon Foam for Cobalt Hydroxide Nanorods Based High Performance Supercapacitor

**DOI:** 10.1038/srep35490

**Published:** 2016-10-20

**Authors:** Umakant M. Patil, Ravindra V. Ghorpade, Min Sik Nam, Archana C. Nalawade, Sangrae Lee, Haksoo Han, Seong Chan Jun

**Affiliations:** 1 Nano ElectroMechanical Device Laboratory, School of Mechanical Engineering, Yonsei University, Seoul 120-749, Republic of Korea; 2 Electronic Material Laboratory, Department of Chemical and Biomolecular Engineering, Yonsei University, Seoul 120-749, Republic of Korea; 3 Chemical Engineering and Process Development Division, CSIR-National Chemical Laboratory, Pune-411008, India

## Abstract

The current paper describes enhanced electrochemical capacitive performance of chemically grown Cobalt hydroxide (Co(OH)_2_) nanorods (NRs) decorated porous three dimensional graphitic carbon foam (Co(OH)_2_/3D GCF) as a supercapacitor electrode. Freestanding 3D porous GCF is prepared by carbonizing, high internal phase emulsion (HIPE) polymerized styrene and divinylbenzene. The PolyHIPE was sulfonated and carbonized at temperature up to 850 °C to obtain graphitic 3D carbon foam with high surface area (389 m^2^ g^−1^) having open voids (14 μm) interconnected by windows (4 μm) in monolithic form. Moreover, entangled Co(OH)_2_ NRs are anchored on 3D GCF electrodes by using a facile chemical bath deposition (CBD) method. The wide porous structure with high specific surface area (520 m^2^ g^−1^) access offered by the interconnected 3D GCF along with Co(OH)_2_ NRs morphology, displays ultrahigh specific capacitance, specific energy and power. The Co(OH)_2_/3D GCF electrode exhibits maximum specific capacitance about ~1235 F g^−1^ at ~1 A g^−1^ charge-discharge current density, in 1 M aqueous KOH solution. These results endorse potential applicability of Co(OH)_2_/3D GCF electrode in supercapacitors and signifies that, the porous GCF is a proficient 3D freestanding framework for loading pseudocapacitive nanostructured materials.

Among existing energy-storage devices, supercapacitors are quite attractive, that provides higher power density than batteries or fuel cells and much higher energy density compared to capacitors^1^,^2^,^3^. Supercapacitors store electric energy through charge accumulation in the electric double layer (EDLCs), or redox reactions (Pseudocapacitive) involving delocalized electrons, or a combination of the two^4^,^5^. Carbon materials such as, activated carbon, carbon nanotubes, graphene nanosheets, etc., have been used in commercial EDLC supercapacitors because of their high specific surface area, thermal stability, good conductivity, and excellent corrosion resistance to electrolytes^6^,^7^,^8^. However, their low specific capacitance (SC), cannot meet the emerging requirements for devices that need high energy and power^9^. Consequently, there has been growing interest for pseudocapacitive materials in supercapacitors by means of high energy density, which is larger at least by one magnitude than EDLCs^10^,^11^.

The energy storage materials with multiple oxidation states, that undergo excellent redox reactions, such as, transition metal hydroxides/oxides and conductive polymers, are considered as pseudocapacitive materials (PCMs)^12^. The cobalt hydroxide [Co(OH)_2_] is one of the best candidates as a PCMs, among many metal oxides/hydroxides, with very high theoretical specific capacitance (3500 F g^−1^), and characteristic redox reactions along with its abundance in nature at low cost^13^,^14^,^15^,^16^. However, obstacles like, poor electrical conductivity and structural degradation of Co(OH)_2_ during redox process, confines to achieve theoretical supercapacitive performance^17^. In the efforts to improve electrochemical performance of Co(OH)_2_ in supercapacitors, various nanostructures and its composites with various carbon materials have been studied and reported^15^,^18^,^19^. Nevertheless, such composites suffers from serious aggregation, which causes inferior accessibility to electrolyte ions and complexed charge transport. So, freestanding, conducting porous carbon frameworks gaining much attention as backbone in energy-storage devices^20^,^21^. Nowadays, lightweight 3D ultrathin graphite foam (UGF) prepared by CVD method or Graphene sponge by chemical methods, have been used as conductive backbone in supercapacitors due to their unique inherent properties like porous structure with high surface area, which provide space for the transportation or storage of electrons and electrolytic ions^22^,^23^. Being lightweight (~20 mg cm^−3^) and continuous conducting structure, make UGFs promising candidate as a substitute for conventional current collectors such as nickel foams (NF) (~262 mg cm^−3^) or carbon paper in energy storage devices^24^,^25^. Despite several advantages of GFs, low conductivity (Graphene sponge) and too much wide porous structure (~200 μm for UGFs) are foremost hurdles for high electrochemical performance^24^.

So, the PolyHIPE derived graphitic carbon foams (GCFs) with controlled porous structure can be the alternative for contemporary GFs or GS. Polymerized high internal phase emulsion (PolyHIPEs) are very attractive for a wide variety of applications, such as catalysis, chromatography, separation, absorption, ion exchange, insulation, tissue engineering, drug delivery, heavy metal separation^26^,^27^,^28^,^29^,^30^,^31^,^32^,^33^,^34^. The HIPEs provide a very convenient route to synthesize macroporous polymer materials by polymerizing monomers in the continuous phase. A HIPE is an emulsion with an internal phase volume greater than 74%, which is the maximum volume that may be occupied by uniform spheres. In a HIPE, the continuous phase forms thin walls around polyhedral droplets of dispersed phase. Polymerization of a continuous phase comprising suitable monomers, followed by removal of the internal phase and surfactant, gives a highly porous polymer called polyHIPE with a structure of cages interconnected by windows^27^. Moreover, this synthetic pathway to produce porous polymers gives the opportunity to easily balance between porosity, functionality and conductivity of final material, offering a great adaptability towards the intended applications^28^. Previously it has been reported that, a porous carbon with high surface area can be obtained by carbonization of a styrene based polymers prepared from a HIPE template^26^,^29^,^30^,^31^,^32^,^33^,^34^. Similar to the GFs, hierarchically porous carbonized polymeric materials have attracted the attention of researchers due to their controlled porous structure and facile synthesis.

On the basis of above considerations, here we report a fabrication of binder-free Co(OH)_2_/GCF hybrid electrode for supercapacitor application. The sulfonated porous polyHIPE were carbonized in an inert atmosphere at temperature up to 850 °C to yield monolithic 3D carbon structures with more than 90% interconnected void space. In polyHIPE synthesis, besides the thermal polymerization, HIPE polymerization is carried out on styrene with divinylbenzene (DVB) as cross-linker using redox initiator system with advantage of short induction period at milder conditions. Moreover, a binder free approach is adopted for fabrication of composite electrode by direct growth of Co(OH)_2_ nanorods on GCF surface using chemical bath deposition (CBD) method. In this manuscript, we educed for the first time a supercapacitor performance of a light weight chemically decorated Co(OH)_2_ nanorods GCF electrode.

## Result and Discussion

### Formation of Co(OH)_2_ Nanorods on GCF

**Figure 1:**
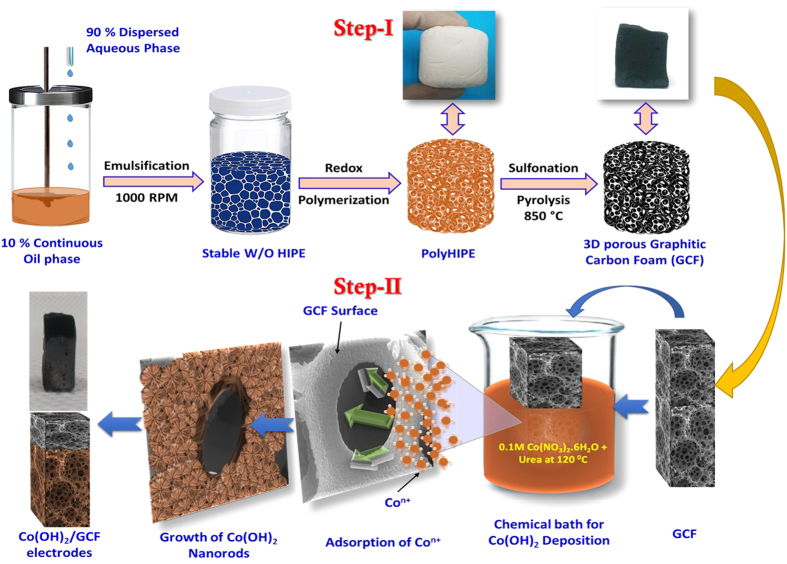
Schematic for preparation of PolyHIPE derived 3D carbon foam, with photographs of PolyHIPE and carbonized 3D GCF, and direct growth of Co(OH)_2_ Nanorods on 3D GCF surface by using facile chemical bath deposition (CBD) method, with actual photograph of prepared Co(OH)_2_/GCF electrode.

For the growth of Co(OH)_2_ over 3D GCF, the 3D porous GCF were prepared by carbonizing polyHIPE at 850 °C. Initially, polyHIPE was synthesized by copolymerizing styrene and divinyl benzene on the water-in-oil HIPE template stabilized by span 80 and to account fabrication of GCF, the schematic is shown in Fig. 1 (step 1) (and reaction scheme given in Figure S1 (See ESI)). In synthesis, redox initiated polymerization is used at moderate temperature (50 °C) and locus of aqueous phase by using water soluble initiator APS as an oxidizing agent with sodium metabisulphite as an accelerator. After polymerization, removal of aqueous phase yielded highly porous polymer having interconnected open porous architecture with open-celled cages called voids interconnected by windows. Sulfonation of polyHIPE with concentrated sulfuric acid is much more effective to create strongly interacting sulfonate moieties in order to stabilize the polymer through initial stages of carbonization and also activates surface of polyHIPE to generate mesopores and micropores on walls of 3D carbon foam^32^. The FTIR spectrum (Figure S2, See ESI) of polyHIPE is agreement with the polymer structure as expected and confirms sulfonation process of the PolyHIPE, qualitatively. Sulfonated polyHIPE shows absorption at 1035 cm^−1^ results from the symmetric stretching vibration of -SO_3_H groups and the absorption at 1126 cm^−1^ results from a sulfonate anion attached to a phenyl ring as well as aromatic -C-H stretching observed at 3027 cm^−1^ in polyHIPE is reduced after sulfonation. To determine carbonization yield and behavior, TGA analysis of polyHIPE and sulfonated polyHIPE were done and given in Figure S3 (See ESI). The weight losses at below 100 °C represent evaporation of imbibed water. PolyHIPE sample shows essentially no weight loss up to 300 °C; then it decrease rapidly between 300 and 470 °C followed by slow losses up to 850 °C. The pyrolysis of polyHIPE is apparently a one-step reaction, whereas that of sulfonated polyHIPE proceeds in two steps: a low-temperature process at 310–500 °C and a high temperature process at 500–580 °C. An important feature with the sulfonated polyHIPE is remarkably higher carbonization yield than that of normal polyHIPE.

Growth of Co(OH)_2_ on GCF was achieved by using facile aqueous CBD method. Since, the surface of carbon foam reveals a certain amount of hydrophobicity, therefore depositing Co(OH)_2_ nanostructure by CBD method from aqueous solutions is slightly challenging. So, different strategies have been employed such as, functionalization of carbon surface and vacuum soaking, in order to enhance wettability of carbon foam^25^,^34^. Surface of carbon foam is treated by H_2_O_2_ to increase the wettability of the GCF surface. This simple strategy facilitates the insinuation of solution from chemical bath into porous structure of carbon foam and ensures further growth of nanostructure. The CBD method, established on construction of a solid phase from solution, which involves nucleation, coalescence and particle growth like collective steps, and one of the efficient “bottom-up” approaches to grow nanostructures^35^. A probable formation mechanism of Co(OH)_2_ over 3D GCF in the presence of urea is illustrated by scheme shown in Fig. 1 (step 2). Under chemical reaction conditions at 90 °C, urea decomposes slowly in to NH_3_ and CO_2_ followed by their hydrolysis producing CO_3_^2−^ and OH^−^ ions (Reaction 1–3)^36^. As time drives, solution becomes more alkaline with the release of NH_3_ accompanied with relatively low soluble CO_2_ in aqueous solution. The reactions lead to consumption of H^+^ and hence increase the pH of the medium. Under these alkaline condition Co^2+^ from Co(NO_3_)_2_, is expected to form its hydroxide as described in reaction 4. Such alkalescent conditions favors heterogeneous nucleation of Co^n+^ ions (Co^2+^ or Co^3+^) on the heterogeneous surface of substrate by electrostatic or van der Waals forces^37^. The reactions involved in the system are expressed as follows.




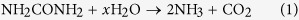







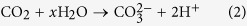







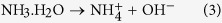







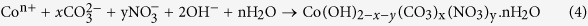




In formation of Co(OH)_2_, the OH^−^ ions are adsorbed on Co^n+^ exposing facets, which enhance the polarity of negatively charged cobalt hydroxide with intercalation of free nitrate and carbonate ions in the van der waal’s gap between the hydroxide. Consequently, dipolar interactions can occur leading to form 2D nanorods like structure by self-assembly via electrostatic force and hydrogen bonding. Accordingly, pink colored Co(OH)_2_ (photograph shown in Fig. 1) is fastened on GCF surface.

### Structural studies

**Figure 2:**
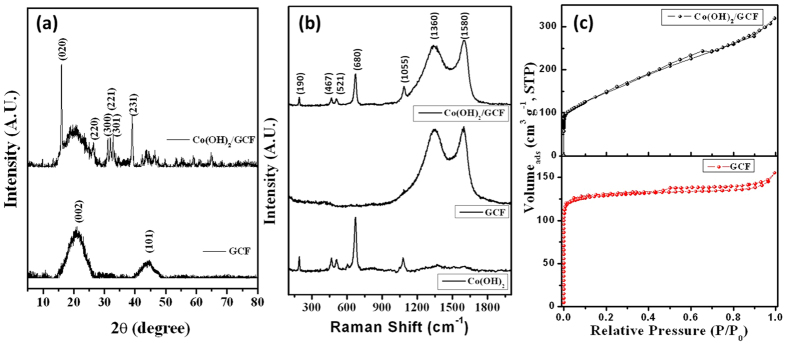
(**a**) The XRD pattern of 3D GCF and Co(OH)_2_/GCF electrodes, (**b**) Raman spectra of 3D GCF, Co(OH)_2_ and Co(OH)_2_/GCF electrodes. (**c**) N_2_ adsorption-desorption isotherms of GCF and Co(OH)_2_/GCF electrodes.

Crystallinity of GCF and Co(OH)_2_/GCF electrodes were analyzed using X-ray diffraction and XRD profiles are depicted in Fig. 2(a). Two significant broad diffraction humps with peaks (2θ) centered at 21.2 and 45.6° are attributed to (002) and (101) reflections of crystalline peak from hexagonal graphite carbon (JCPDS: 75–1621)^38^. The XRD pattern of Co(OH)_2_/GCF reveals that, characteristic peaks at ~16.01, 27.6, 33.2, 34, 34.4, and 39.1° corresponds to the (020), (220), (300), (221), (301), and (231) diffraction planes, respectively and it can be indexed as cobalt nitrate carbonate hydroxide, which are consistent with results in literature or standard card (JCPDS Card No. 38–0547)^13^,^16^. It is well known that, Co(OH)_2_ is isostructural with hydrotalcite-like compounds and consist of stacked Co(OH)_2-x_ layers intercalated with various anions (e.g., nitrate, carbonate, hydrate etc.) in interlayer space to restore charge neutrality and can be termed as cobalt hydroxide nitrate hydrate [Co(OH)_2-x-y_(CO_3_)_x_(NO_3_)_y_.nH_2_O]^39^,^40^. In general, according to intercalated anions, cobalt hydroxide can be crystallized into a hexagonal layered structure with two (α and β) polymorphs. The α phase has a larger interlayer spacing (7.0 Å) than the brucite-like β phase (4.6 Å), large interlayer distance can result in to high electrochemical capacitive performance^40^. The prepared Co(OH)_2_ shows interlayer spacing about ~5.5 Å indicating probably formation of mixed phase (α and β) on 3D GCF surface.

Raman analysis is performed to determine the feature of prepared GCF, Co(OH)_2_, and Co(OH)_2_/GCF electrodes and presented in Fig. 2(b). The two obvious peaks centered at approximately at 1580 and 1360 cm^−1^ can be seen for GCF and Co(OH)_2_/GCF electrodes, which are belongs to the G-band and D-band, respectively^41^. In Raman spectra of Co(OH)_2_, five broad peaks at 190, 467, 521, 680 and 1055 cm^−1^ are observed^42^,^43^. The Raman spectrum of prepared Co(OH)_2_ is dominated by an intense highly polarized band at 680 cm^−1^ and attributed to CoO (A1g) symmetric stretching mode. The doublet peak at 467 and 521 cm^−1^, can be attributed to an O–Co–O bending and CoO(Ag) symmetric stretching mode. The initial band at 195 cm^−1^ is attributed to F2g mode. Moreover, the peak at 1042 cm^−1^, can be assigned to the OH^−^ deformation mode, such deformation bands are normally of low intensity in Raman spectra^44^. Analogous, five peaks originated from Co(OH)_2_, with low intensity along with D and G band, can be seen in Raman spectra for Co(OH)_2_/GCF electrode. Degree of defect for the carbon is measured by the ratio of intensities for D-band and G-band (ID/IG). As seen from Fig. 2(b), the ID/IG ration for pristine CF is found to be 0.97 and decreases to 0.92 for Co(OH)_2_/CF electrode, indicating deposition of Co(OH)_2_ on GCF surface. These obvious intensities of D and G band with ratio up to 1 attributed to partial graphitic structure of prepared carbon foam^41^.

The N_2_ adsorption-desorption isotherms of GCF and Co(OH)_2_/GCF (heat treated at 300 °C, to remove interlayer water molecule, nitrate and carbonate ions) electrodes are presented in Fig. 2(c). The observed isotherms of GCF and Co(OH)_2_/GCF electrodes can be classified as Brunauer–Deming–Deming–Teller (BDDT)- isotherm type (II) with hysteresis H4-type in the IUPAC classification. The isotherms in Fig. 2(c) are type II isotherms, typical of solids having a bimodal distribution of pore widths (micro and mesopores) and reflecting presence of well-ordered wide macropores (voids and windows) sections containing narrow mesopores interconnecting channels^45^,^46^. After complete micropore filling near (P/P0) 0.1, nitrogen uptake monotonically continues with increasing pressure, owing to nitrogen adsorption in the mesoporous structure. The reversible type II isotherm reveals rapid initial amount adsorbed at low pressure followed by flat region. The curve approaches the saturated vapor pressure (P/P0) line asymptotically and the desorption curve may lie above the adsorption curve, and it is accepted that such shape is characteristic of micropore filling. At the high pressure region, a distinct hysteresis shape type (H4) can be seen for both electrodes. The adsorption branch is steep at saturation pressure, the desorption branch is slopping. These occur for heterogeneous assembly of capillaries with bodies of wide dimensions and having a greatly varying range of narrow neck and wedge shaped capillaries open at both ends^46^. This finding suggesting mesoporous structure of pristine GCF and Co(OH)_2_ deposited GCF electrodes. According to the fitting analysis to Brunauer–Emmett–Teller (BET) equation, the surface areas of electrodes are estimated to be 389 and 520 m^2^ g^−1^ for GCF and Co(OH)_2_/GCF electrodes, respectively. The enhanced surface area of Co(OH)_2_/GCF electrode than pristine GCF highlighting the usefulness of freestanding porous interconnected GCF structure. Conversely, conventional carbon/graphene based composites materials reveals reduction in surface area due to aggregation. The pore size distribution based on Barrett–Joyner–Halenda (BJH) method (Figure S4 in ESI) clearly demonstrates presence of mesopores on carbon wall with an average diameter of about ~5 nm and uneven pores distribution <~10 nm for Co(OH)_2_/GCF electrode, this result can be interpreted as a well-ordered slit-shaped mesopores containing narrow and wide sections by the excess Co(OH)_2_ guest nanorods. The surface area offered by Co(OH)_2_ nanorods improves the surface area of hybrid electrode. Thus, XRD, Raman and BET measurements indicates that, the hydrous Co(OH)_2_ is grown over the partially graphitic carbon foam (GCF) and together could provide large accessible surface area, which play a vital role in enhancing supercapacitive performance.

### Morphological analysis

**Figure 3:**
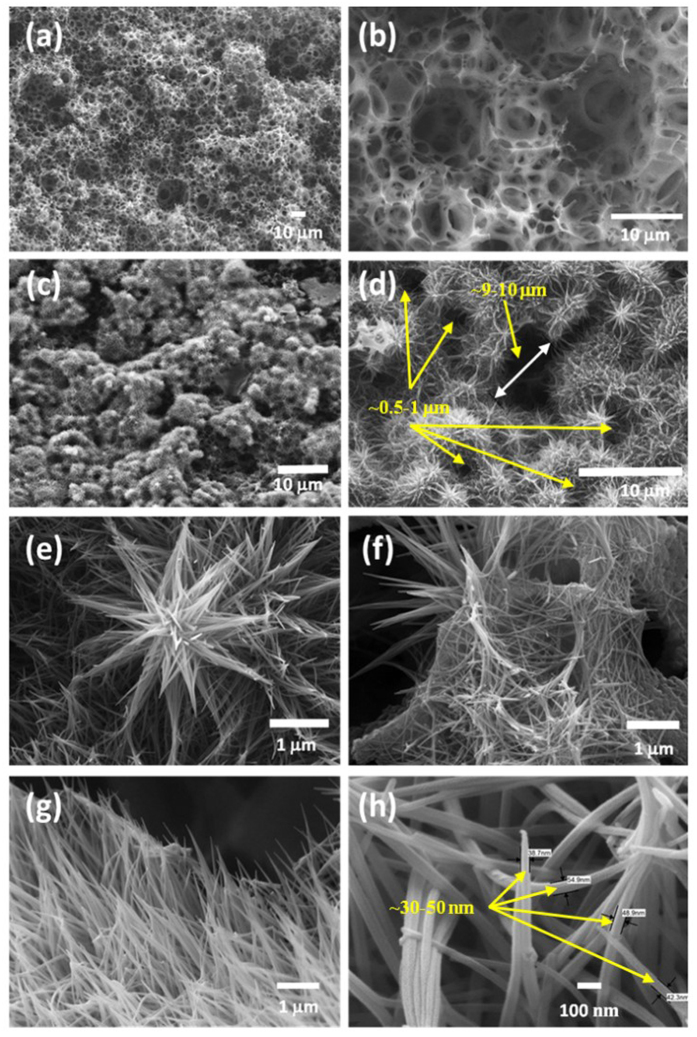
FESEM images of (**a,b**) GCF framework at different magnification, (**c,d**) Co(OH)_2_ microflowers over GCF at different magnifications, (**e–g**) magnified view of overgrown Co(OH)_2_ on surface and edges with (**h**) nanorods at higher magnification.

The SEM micrographs of pristine and Co(OH)_2_ deposited GCF electrodes at different magnifications are shown in Fig. 3. An FESEM image of wide porous pristine 3D carbon framework skeleton at low magnifications can be seen in Fig. 3(a,b). The interconnected GCF electrode retains the 3D structure like monolithic polyHIPE template without much disruption as shown in Figure S5 (a_1–3_ and b_1–3_) (See ESI). The polyHIPE shows, bimodal pore distribution with interconnected voids (~12–14 μm) consisting windows (~3–4 μm) in diameter (Figure S5 (a_2–3_), See ESI). Such, development of macroporous polymeric foam via a HIPE method, in which the oil phase of emulsion gradually polymerizes into a monolithic, porous polymer, while the aqueous phase, initially serving as liquid droplet template, gradually evaporates, leaving interconnected macrospores behind. After sulfonation and carbonization of polyHIPE, formed carbon foam (shown in Figure S5 (b_2_), See ESI) retain its analogous macro porous structure with void size about ~14 μm having windows of ~4 μm. The width of individual stems of carbon foam is found to be around ~1–5 μm (shown in Figure S5 (b_3_), see ESI). A smooth and thin carbon skeleton with 3D porous structure (Fig. 3(b)) and structure of carbon is well remained without any cracks, demonstrating a continuous network of carbon framework. A wide porous carbon foam skeleton is well-decorated by Co(OH)_2_, can be seen at a low magnification SEM image shown in Fig. 3(c). The twigs of carbon foam are well-covered with overgrown Co(OH)_2_ microflowers (Fig. 3(c,d)). Nucleation and coalescence process during growth of Co(OH)_2_ by CBD method can be inveterate by overgrowth of material. After growth of Co(OH)_2_ microflowers voids reduced up to ~9–10 μm and windows reduced up to ~0.5 to 1 μm revealed in Fig. 3(d). The SEM images of Co(OH)_2_ microflower grown at different positions over GCF surface shown in Fig. 3(e–g). The SEM image shown in Fig. 3(e) revels that, the Co(OH)_2_ microflowers are in averagely 3–4 μm in size and comprises with centrically grown nanorods. However, at edges of GCF (shown in Fig. 3(f,g)), randomly grown Co(OH)_2_ nanorods can be seen instead of microflowers. The high magnification SEM image (Fig. 3(h)) demonstrates that, these Co(OH)_2_ nanorods are ~1–2 μm in length with width of ~30–50 nm. Such, nanorod-like morphology leads to a high specific surface area with better diffusion paths, which may provide structural foundation for the high specific capacitance^44^. Furthermore, SEM-EDS analysis (Figure S6) was carried out to find particulate deposited elements and interconnection of Co(OH)_2_ nanorods with the GCF surface. The EDS analysis shows that, 19.2% cobalt, 21.4% oxygen and 30% of carbon originated from GCF demonstrates formation of Co(OH)_2_/GCF composite electrode. The 3D porous structure of GCF and Co(OH)_2_ nanorods synergistically may provide large accessible surface area and subsequently enhancing the supercapacitive performance.

### Supercapacitive performance

**Figure 4:**
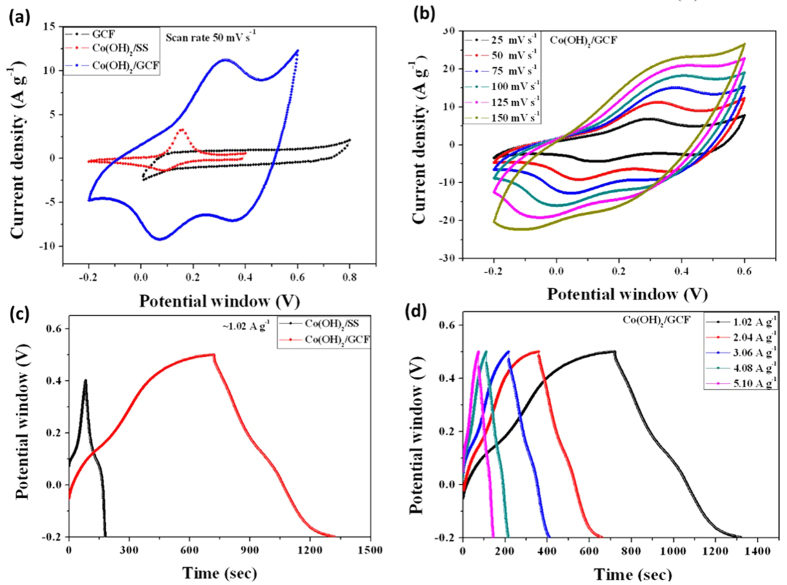
Electrochemical studies (**a**) CV curves of Co(OH)_2_/GCF, Co(OH)_2_/SS and GCF electrodes within optimized potential window of −0.2 to 0.6 V, −0.2 to 0.4 V and 0.0 to 0.8 V, respectively, in aqueous 1 M KOH at 50 mV s^−1^ scan rate. (**b**) Scan rate dependent (25–150 mV s^−1^) CV curves of Co(OH)_2_/GCF electrode in 1 M KOH electrolyte. (**c**) Galvanostatic charge-discharge (GCD) plots of Co(OH)_2_/GCF and Co(OH)_2_/SS electrodes within potential window of −0.2 to 0.4 V and −0.2 to 0.5 V, respectively, at constant charging current from ~1 A g^−1^. (**d**) GCD plots of Co(OH)_2_/GCF electrode at different charging-discharging current densities from ~1 to 5 A g^−1^.

The supercapacitive performances of Co(OH)_2_/GCF electrodes were tested by forming a half-test cell in 1 M KOH aqueous electrolyte. Herein, the 3D porous GCF is castoff as a current collector backbone, without any use of carbon black additive and binder, for directly grown Co(OH)_2_ nanorods. For comparison, CBD deposited Co(OH)_2_ on stainless steel (SS) substrate were used as conventional supercapacitor electrode. The cyclic voltammogram (CV) curve is an ideal tool for characterizing and assure capacitive features in terms of magnitude of current and shape of voltammogram. The Fig. 4(a) shows, the CV curves at 50 mV s^−1^ for Co(OH)_2_/SS, Co(OH)_2_/GCF and GCF electrodes. It is noticeable that, the CV curves of Co(OH)_2_, either on SS or GCF electrode, exhibit two intense characteristic redox peaks arising from the reversible faradaic reaction in KOH electrolyte. For Co(OH)_2_/SS electrode, the anodic peak (+ve current density) observed at +0.15 V corresponds to an oxidation reaction Co(OH)_2_ to CoOOH, while cathodic peak (−ve current density) occurred around +0.04 V indicates the reverse process, i.e., a reduction reaction. However, Co(OH)_2_/GCF electrode, shows shift in redox peak position as, anodic and cathodic peak at +0.23 and +0.03 V, respectively. Such shift can be ascribed to formation of different electrochemical layers over dissimilar surfaces of substrates. Herein, two parallel mechanisms may involve in charge storage, based on adsorption and intercalation involving surface and bulk phenomena during the Redox reaction in porous Co(OH)_2_/GCF electrodes. The pseudocapacitive performance for Co(OH)_2_/GCF electrode based on a redox mechanism can be explained as follows:




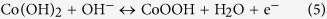




As usual pristine GCF shows rectangular shaped CV, in 0 to +0.8 V potential window, features typical electrochemical double layer capacitive (EDLC) behavior like other carbon based materials^47^. Figure 4(a) reveals that, current under curve of CV is higher for Co(OH)_2_/GCF electrode than Co(OH)_2_/SS and GCF electrodes, which may be due to its larger specific surface area and easy intercalation and de-intercalation reaction of ions involved in basic electrolyte. The Fig. 4(b) shows the CV curves at different scan rates ranging from 25 to 150 mV s^−1^ for Co(OH)_2_/GCF (for GCF and Co(OH)_2_/SS electrodes shown in Figure S7(a,b), See ESI). The current under curve increasing with scan rate for all electrodes; concludes that voltammetric current is directly proportional to scan rates of CV, indicating electrochemical reaction of Co(OH)_2_/GCF in electrolyte is a diffusion-controlled process which signifies ideal capacitive behavior.

**Figure 5:**
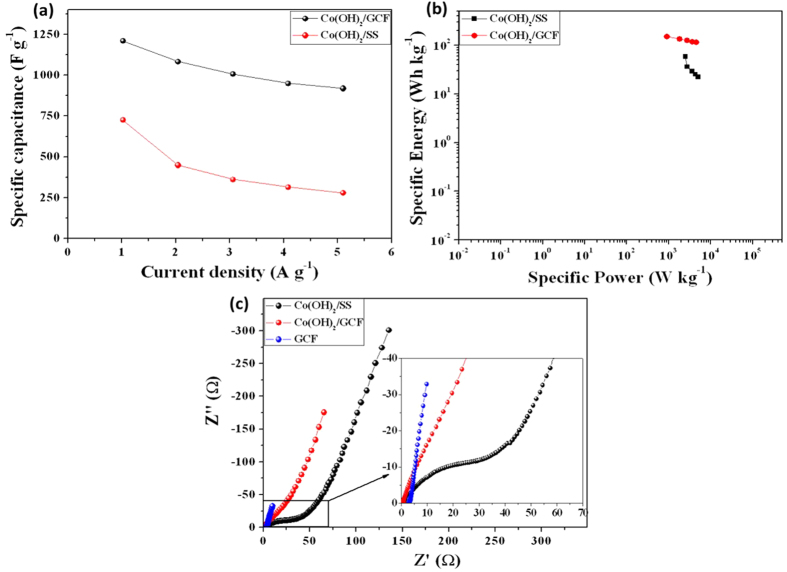
(**a**) Graph of charge-discharge current dependent specific capacitances. (**b**) Typical Ragone plot of specific energy and power for Co(OH)_2_/GCF and Co(OH)_2_/SS electrodes. (**c**) Electrochemical impedance spectrum within 1 MHz to 10 mHz frequency region, a Nyquist plot of GCF, Co(OH)_2_/GCF and Co(OH)_2_/SS electrodes. Inset shows enlarged Nyquist plot at higher frequency.

**Figure 6:**
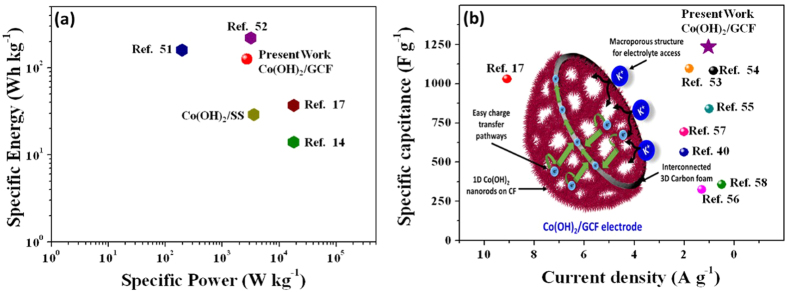
Comparative (**a**) Ragone plot of specific energy and power, (**b**) specific capacitance at ~1 A g^−1^ for Co(OH)_2_/GCF electrode with other reported references and inset of figure shows schematic of charge storage and fast charge transfer mechanism in Co(OH)_2_/GCF electrode.

Galvanostatic charge/discharge (GCD) measurements were measured at ~1 A g^−1^ current density and shown in Fig. 4(c). The shape of GCD curves are different from EDLC based capacitance (ideally linear), which is analogous with the result of CV analysis. From GCD plots, it is observed that (i) non-linear shape extant for Co(OH)_2_/SS and Co(OH)_2_/GCF electrodes, indicating that the capacity results mainly from a pseudocapacitive mechanism (according to reaction 4). Wherever, the redox reaction for Co(OH)_2_ electrode is usually considered as an intercalation and de-intercalation of charges into and out of the bulk of the solid phase. (ii) The Co(OH)_2_/GCF composite electrode delivers a very wide charge-discharge time compared with little linearity than for Co(OH)_2_/SS electrode. (iii) It is noteworthy that, because of GCF potential window of Co(OH)_2_ increases by 0.1 V and such remunerations may provide by porous structure with well-spaced coaxial geometry of Co(OH)_2_ over GCF twigs. The Fig. 4(d) shows, GCD plots of Co(OH)_2_/GCF at different constant current densities from ~1 to 5 A g^−1^ (for Co(OH)_2_/SS shown in Figure S8, See ESI). The specific capacitance is calculated from GCD curves, at different charge-discharge current densities and shown in Fig. 5(a). The graph reveals that, specific capacitance decreases with increase in GCD current densities. This result indicating that, at higher charging rates, a surface confined redox process may occur, which specifies the limitation arising from the charge transfer kinetics^47^. The decreasing specific capacitance is consequence of less active material access, which reduces the effective utilization of material at a higher charging-discharging rate. Consequently, specific capacitance obtained at slowest scan rate is believed to be closest to that of full utilization of electrode material^48^. Maximum specific capacitances is found to be ~1235 and ~720 F g^−1^ for Co(OH)_2_/GCF and Co(OH)_2_/SS electrodes, respectively, at ~1 A g^−1^. The specific capacitance of Co(OH)_2_/GCF is significantly higher than that of Co(OH)_2_/SS electrode. A distinctive Ragone plot is shown in Fig. 5(b) with the corresponding specific energy and power with current densities of ~1 to 5 A g^−1^. The maximum specific power and energy for Co(OH)_2_/GCF and Co(OH)_2_/SS electrode are found to be 2.7, 2.5 kW kg^−1^ and 125, 58 Wh kg^−1^, respectively. For better understanding the enormous benefits of 3D carbon framework in supercapacitors, EIS measurements were carried out. The EIS is measured for Co(OH)_2_/GCF, Co(OH)_2_/SS and GCF electrodes at open circuit potential (OCP) over the frequency range of 10 MHz and 10 mHz and compared in Fig. 5(c). A sharp increase in Zim at lower frequency, attributed to capacitive behavior of material and it can be seen for all three electrodes. At higher frequencies, a semicircle is observed only for Co(OH)_2_/SS electrodes, that implies for charge transfer resistance (R_ct_) at electrode/electrolyte interface, and inclined impedance line found for Co(OH)_2_/GCF electrode, except for pristine GCF electrode. For an ideal double-layer capacitor, the impedance plot should be a vertical line, parallel to imaginary axis and notably GCF electrode shows almost parallel line to imaginary impedance (shown in inset of Fig. 5(c)), which is generally observed for carbon-based materials such as activated carbon, graphite, CNTs, and graphene^49^. The “R_ct_” can be calculated from the radius of the initial curvature at higher frequencies^50^. The inclined impedance line at higher frequencies offers negligible “R_ct_” for Co(OH)_2_/GCF and GCF electrodes than that of the Co(OH)_2_/SS (~25 Ω) electrode. As shown in inset of Fig. 5(c), solution resistance (R_s_) of Co(OH)_2_/GCF electrode (0.45 Ω) is much smaller than that of Co(OH)_2_/SS electrode (1.7 Ω) and GCF (2.9 Ω) electrode. This results signifies that, GCF-based Co(OH)_2_ electrode possesses lower contact and charge-transfer resistance. Conversely, high “R_ct_” and “R_s_” of Co(OH)_2_/SS electrode restrict it to achieve maximum supercapacitive characteristics. A rough comparison of obtained maximum specific capacitance, and specific energy and power (typical Ragone plot), at particular current density (~1 A g^−1^), with some recent reports for half test cell is shown in Fig. 6(a,b). Figure 6(a) indicates that, the GCF-based Co(OH)_2_ electrode possesses moderate specific energy and power compared with previous literature values reported for aqueous electrolyte based Co(OH)_2_ on different substrate^14^,^17^,^51^,^52^. The obtained maximum specific capacitance is higher than that reported for Co(OH)_2_ with various nanostructures on different substrate and graphene-Co(OH)_2_ composites^17^,^40^,^53^,^54^,^55^,^56^,^57^,^58^, as shown in Fig. 6(b), with the exception of chlorine doped Co(OH)_2_ (9894 F g^−1^) on the Ni foam electrode^52^.

## Discussion

The improved synergistic interaction between GCF and Co(OH)_2_ ensures the superior capacitive characteristic compared to conventional Co(OH)_2_ on SS substrate. The Co(OH)_2_/GCF electrode with high surface area (520 m^2^ g^−1^) offers many advantages over conventionally used Co(OH)_2_/SS and Co(OH)_2_ based composite electrodes in supercapacitors. Based on the outcomes, superiority of 3D GCF framework for Co(OH)_2_ over conventional electrodes such as metal current collectors (SS), graphene based composites, nickel foam (NF), graphene foam (GF) etc., is discussed in brief as, (i) the freestanding 3D porous network of GCF provides a large surface area (389 m^2^ g^−1^) and easy access to grow nanostructures. Also, porous structure provides informal admittance for electrolytic ions inside well-organized porous matrix of Co(OH)_2_/GCF, reduces the transport length of ions inside the nanochannels, and hence decreases diffusion resistance (schematic shown in inset of Fig. 6(b)). Conversely, the Co(OH)_2_/SS or conventional composite electrode suffer from higher ion diffusion resistance due to compact structure or aggregation during composite formation. (ii) In conventional supercapacitors, the weight of conductive substrates (NF or SS) accounts for a large mass portion in the electrode as compared to lightweight GCF (23 mg cm^−3^)^24^. (iii) Conventional Co(OH)_2_/SS or resistive binder enriched, loosely interconnected nanostructures of Co(OH)_2_ or Graphene- Co(OH)_2_ composites (at high thickness), offers self-dimensional restrictions at charge transportation during charge-discharge. However, 3D GCF serves as excellent backbone to Co(OH)_2_ nanorods with high surface area and easy charge transportation during the charge-discharge process (schematic shown in inset of Fig. 6(b))^23^,^24^. And iv) finally, maximum utilization of 3D GCF electrodes space by means of competitive bimodal porous distribution with voids (~14 μm) and windows (~4 μm), unlike the popular wide porous (~200 μm) 3D graphene foam (3D UGF) or NF^21^,^22^,^23^. As demonstrated here, GCF is uniquely advantageous to assist as a 3D support of large capacity and high specific energy to uniformly anchored Co(OH)_2_ NRs. Hence, herein we presented effective utilization of chemically synthesized 3D GCF for growth of Co(OH)_2_ NRs as exceptional electrode for supercapacitor.

In summary, the 3D graphitic carbon network, with hierarchical porous structure, was fabricated from polyHIPE template by carbonizing at 850 °C. Moreover, a facile CBD method was successfully employed for direct growth of Co(OH)_2_ nanorods (width 30–50 nm) onto the 3D GCF surface for supercapacitor application. Resultant, Co(OH)_2_/GCF electrode with enhanced surface area (520 m^2^ g^−1^) exhibits a maximum specific capacitance of up to ~1235 F g^−1^. The improved supercapacitive performance of Co(OH)_2_/GCF emanates from the synergistic co-operation between 3D graphitic carbon foam and Co(OH)_2_ nanorods, leading to high specific power and energy of 2.7 kW kg^−1^ and 125 Wh kg^−1^, respectively. Convincingly we believe that, the facile synthetic approaches, such as CBD and polyHIPE template, provides a convenient route for preparation of Co(OH)_2_ nanorods over 3D GCF as an efficient electrode for high energy storage applications and this work will evoke a new opening scope in asymmetric, hybrid, non-aqueous and solid state supercapacitive devices as per the necessity (high energy or power) of applications.

## Methods

### Materials

The monomer and cross-linking comonomer were styrene (Aldrich) and DVB (containing 20% ethylstyrene, Aldrich). Styrene and DVB were washed to remove the inhibitor with a 5 wt% sodium hydroxide aqueous solution and then deionized water. The surfactant used to stabilize the HIPE was sorbitan monooleate (Span 80, Aldrich). A stabilizing salt, calcium chloride [CaCl_2_.6H_2_O, Aldrich], was added to the aqueous phase. The redox initiators were ammonium persulphate (APS) (Merck, India) and sodium metabisulphite (Aldrich). For Co(OH)_2_ synthesis, cobalt nitrate [Co(NO_3_)_2_.6H_2_O] (Sigma-Aldrich USA) and urea [Co(NH_2_)_2_] (Sigma-Aldrich USA) were used as precursors.

### Preparation of polyHIPE derived 3D-Graphitic carbon foam

The HIPEs are normally prepared by a mixing of a continuous phase and a large proportion of internal phase with vigorous stirring in the presence of a suitable surfactant. The continuous phase undergoes polymerization, subsequently after removal of disperse phase a highly porous polymer can be obtained. Finally the polymer is functionalized and pyrolyzed at high temperature to yield free standing 3D carbon network. In a typical experiment, aqueous phase containing calcium chloride (0.09 g) was added to the continuous phase containing styrene (0.808 g), DVB (0.101 g), surfactant sorbitan monooleate (Span 80) (0.272 g) and oxidant APS (1 mol% w.r.t. to monomers), drop wise stirring with overhead stirrer having Ruston blades at 1000 rpm. After complete addition of aqueous phase, the emulsion was stirred further 2 minutes and then reducing agent sodium metabisulphite (0.5 mol% w. r. t monomer) was added to the emulsion. Dispersed phase to continuous phase ratio is maintained as 1:10. This emulsion phase was then transferred to a polypropylene mold and kept for polymerization at 50 °C for 8 hours. After polymerization, the monolithic polyHIPE was removed from the mold and washed with distilled water for 24 h then with ethanol for 24 h. The polymer is dried to constant weight in a vacuum oven at 50 °C. Porous polymer network was sulfonated using concentrated sulfuric acid. Polymer was immersed in the acid and kept under vacuum for 30 min, to ensure penetration of the pores by acid, then heated at 95 °C for 10 h. After sulfonation, polymer was removed from the acid, left in air for about two days to allow the acid to become diluted, washed in a Soxhlet extractor with distilled water for 24 h, and then allowed to dry. The sulfonated polyHIPE was then carbonized in alumina boat under a N_2_ atmosphere in a tube furnace at 850 °C.

### Growth of Co(OH)_2_ nanorods on 3D-Graphitic carbon foam

Preparation of Cobalt hydroxide nanostructure by chemical bath deposition CBD method is based on the heating of substrate contained aqueous bath of Cobalt nitrate and Urea precursors. Typically, 0.1 M Co(NO_3_)_2_·6H_2_O and 0.1 M CO(NH_2_)_2_ were dissolved in 50 mL of deionized water with magnetic stirring for 10 min to form a homogeneous solution. A piece of GCF (3 cm × 1 cm × 0.5 cm) was treated with 15% H_2_O_2_ solution in water at 60 °C for 30 min, followed by washing with DI water with repeated times. After drying, CGF was placed vertically, with the support of glass microslide, in the prepared chemical bath for deposition. Then bath was sealed and placed in a vessel containing paraffin oil for constant heating. The temperature of system was maintained, without stirring, at 120 °C for 12 h and then allowed to cool down to room temperature spontaneously. After the reaction, sample was rinsed with distilled water to remove the residual reactants and dried at ambient temperature. The weight of deposited material (~0.98 mg cm^−3^) was determined gravimetrically by measuring change in weight of GCF before and after material deposition. For comparison, a conventional electrode were prepared by depositing Co(OH)_2_ NRs from the same bath on a commercial stainless steel (SS) substrate.

### Characterization

The electrode materials were structurally characterized by XRD, Raman, FESEM, and measurements. The morphology of the composite was examined by field-emission scanning electron microscopy (FESEM, JSM-7001F, JEOL). FTIR spectra were obtained using Excalibur series (DIGILAB Co.) instrument in the range of 400–4000 cm^−1^ using KBR pellets. Thermogravimetric analysis (TGA) was conducted using thermogravimetric analyzer (Q 50, TA Instrument), at a heating rate of 10 °C min^−1^ in nitrogen atmosphere. Raman spectra were recorded at ambient temperature on a WITeck ALPHA300M Raman System (excitation at 532 nm, 2.33 eV). The X-ray diffraction (XRD) was carried out on a Rigaku Ultima diffractometer using Cu-Kα radiation. The N_2_ adsorption/desorption was determined by Brunauer- Emmett-Teller (BET) measurements using Quantachrome instrument (Version 3.01). The individual electrochemical performances of Co(OH)_2_/GCF and Co(OH)_2_/SS electrodes were measured by forming half-test cell. A conventional half-test cell contains three-electrode system comprises with Co(OH)_2_/SS or Co(OH)_2_/GCF as working electrode, Ag/AgCl as reference electrode and platinum (Pt) as counter electrode in a 1 M aqueous KOH electrolyte.

## Additional Information

**How to cite this article**: Patil, U. M. *et al.* PolyHIPE Derived Freestanding 3D Carbon Foam for Cobalt Hydroxide Nanorods Based High Performance Supercapacitor. *Sci. Rep.* **6**, 35490; doi: 10.1038/srep35490 (2016).

## Supplementary Material

Supplementary Information
