# Supplementary material for: PolyHIPE Derived Freestanding 3D Carbon Foam for Cobalt Hydroxide Nanorods Based High Performance Supercapacitor
Source: Sci Rep. 2016 Oct 20;6:35490. doi: 10.1038/srep35490 (PMC5071864; doi:10.1038/srep35490)
Supplement: Supplementary Information [file srep35490-s1.pdf]

Supporting information

PolyHIPE Derived Freestanding 3D Carbon Foam for Cobalt Hydroxide Nanorods Based High Performance Supercapacitor

Umakant M. Patil,[1‡] Ravindra V. Ghorpade,[2‡] Min Sik Nam[1], Archana C. Nalawade[3], Sangrae Lee[2], Haksoo Han[2*] and SeongChan Jun[1*]

[1]Nano ElectroMechanical Device Laboratory, School of Mechanical Engineering, Yonsei University, Seoul 120-749, Republic of Korea.

[2]Electronic Material Laboratory, Department of Chemical and Biomolecular Engineering, Yonsei University, Seoul 120-749, Republic of Korea.

[3]Chemical Engineering and Process Development Division, CSIR-National Chemical Laboratory, Pune-411008, India.

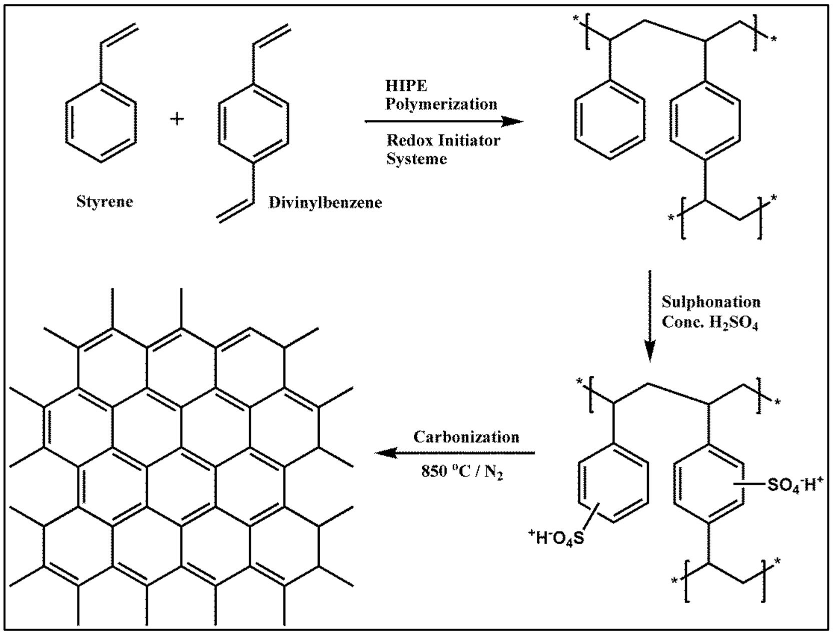

Figure S1 Reaction scheme for PolyHIPE synthesis and carbonization.

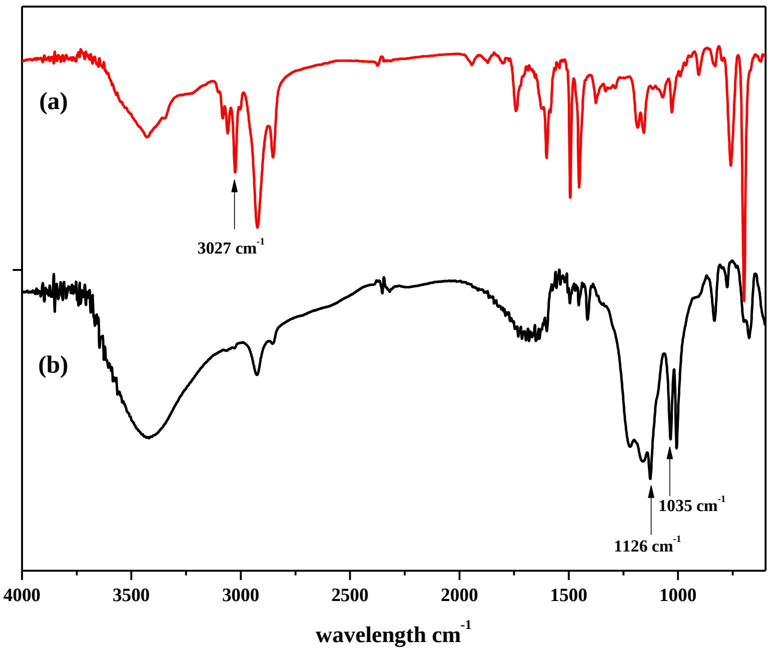

Figure S2 FTIR spectra of (a) polyHIPE and (b) sulfonated polyHIPE. The FTIR spectrum of polyHIPE reveals and confirms sulfonation process of the PolyHIPE, qualitatively. Particularly, sulfonated polyHIPE shows absorption at 1035 cm^{-1} results from the symmetric stretching vibration of $-SO_3H$ groups and the absorption at 1126 cm^{-1} results from a sulfonate anion attached to a phenyl ring as well as aromatic -C-H stretching observed at 3027 cm^{-1} in polyHIPE is reduced after sulfonation.

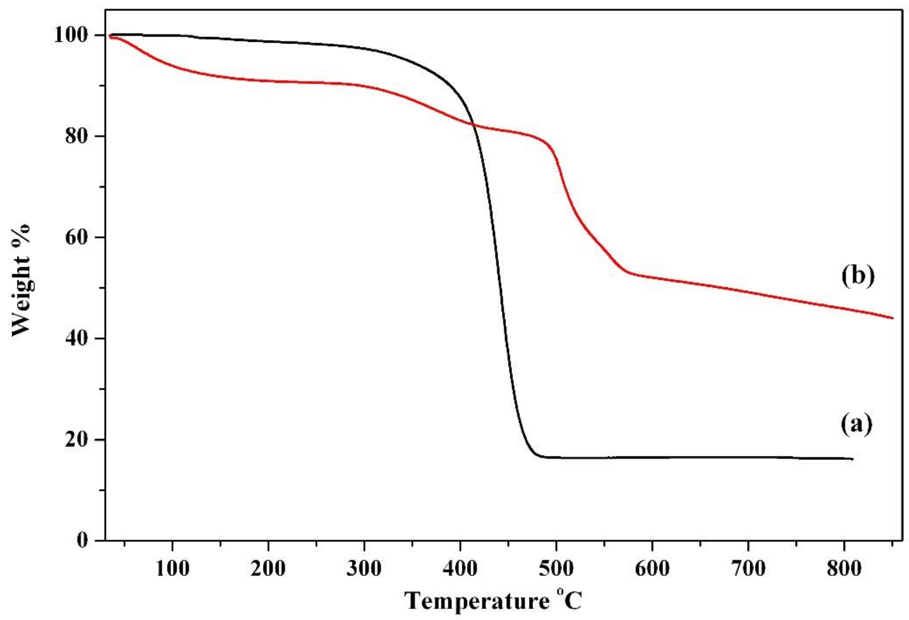

Figure S3 TGA thermograms of (a) polyHIPE and (b) sulfonated polyHIPE. The TGA reveals, weight losses below 100 °C due to the evaporation of imbibed water. Furthermore, no significant weight loss up to 300 °C; then it decrease rapidly between 300 and 470 °C followed by slow losses up to 850 °C. The pyrolysis of polyHIPE is apparently a one-step reaction, whereas that of sulfonated polyHIPE proceeds in two steps: a low-temperature process at 310-500 °C and a high temperature process at 500-580 °C. Prominently TGA reveals that, the sulfonated polyHIPE has remarkable carbonization yield than that of normal polyHIPE.

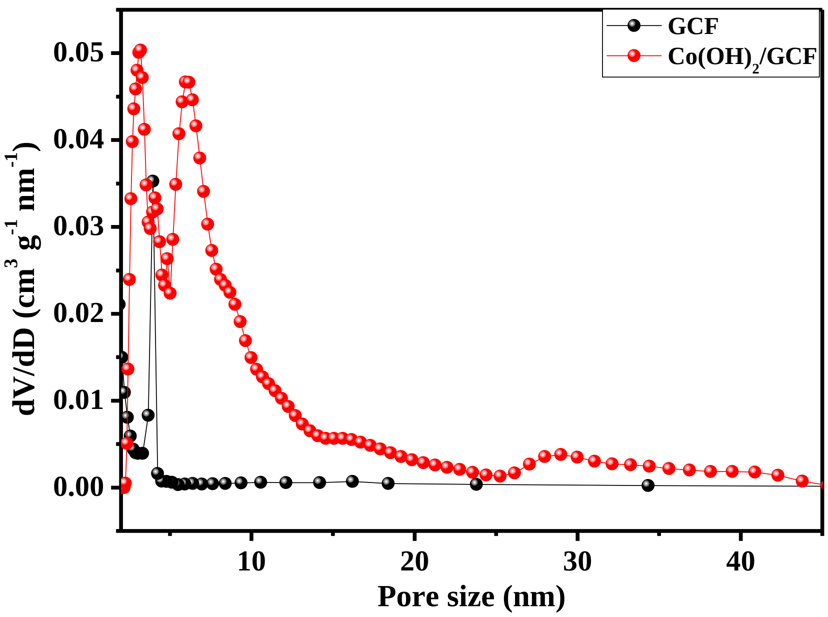

Figure S4 Pore size distribution curves of GCF and $Co(OH)_2$/GCF electrodes. The pore size distribution based on Barrett–Joyner–Halenda (BJH) method, clearly demonstrates presence of mesopores presented by carbon wall with an average diameter of about ~5 nm and uneven pores distribution <~10 nm for $Co(OH)_2$/GCF electrode.

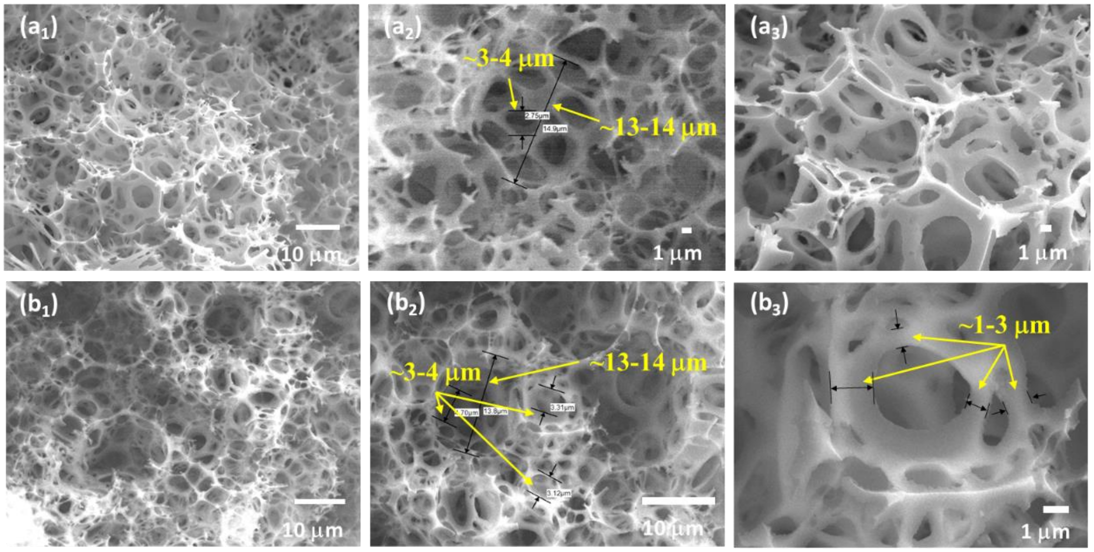

Figure S5 The SEM micrographs of ($a_{1\text{-}3}$) polyHIPE and ($b_{1\text{-}3}$) carbonized polyHIPE (i.e. 3D graphitic carbon foam). The interconnected GCF electrode shown in Fig ($b_{1\text{-}3}$), retains the 3D structure like monolithic polyHIPE template without much disruption (as shown in Fig. ($a_{1\text{-}3}$)). The polyHIPE shown in fig. ($a_{2\text{-}3}$) has bimodal pore distribution with interconnected voids (~13-14 µm) consisting windows (~3-4 µm) in diameter. As shown in figure (b_2), after sulfonation and carbonization of polyHIPE, formed GCF retain its analogous macro porous structure with void size about ~14 µm having windows of ~4 µm with width of individual stems of carbon foam around ~1-5 µm (fig. (b_3)).

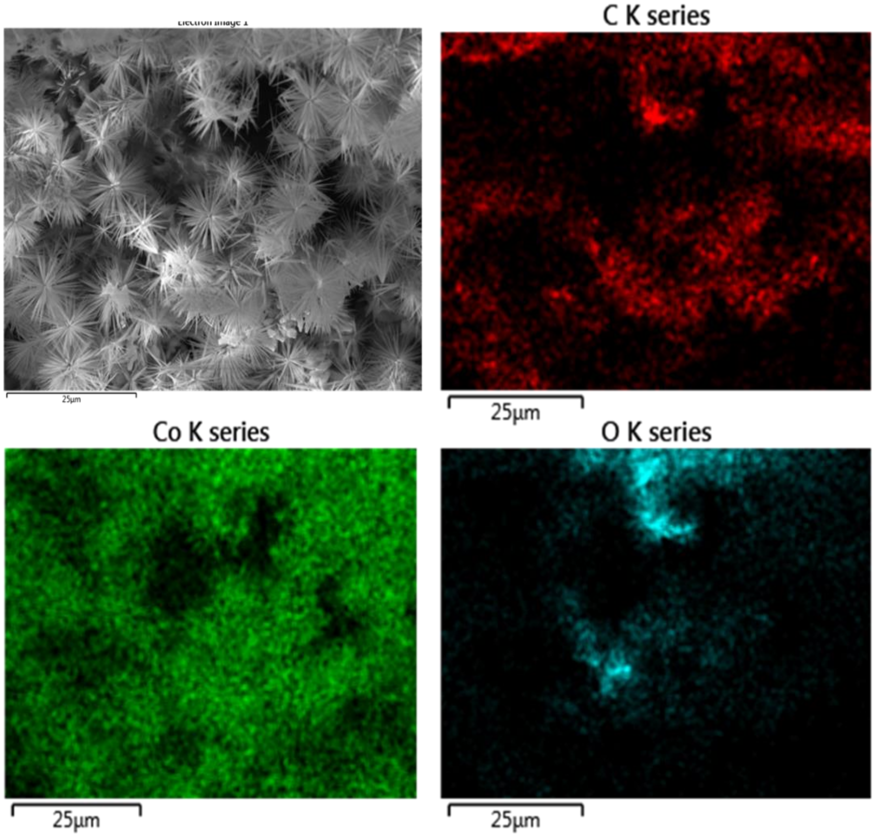

Figure S6 EDS mapping analysis of the $Co(OH)_2$/3D GCF surface with the content of Carbon, Cobalt and Oxygen.

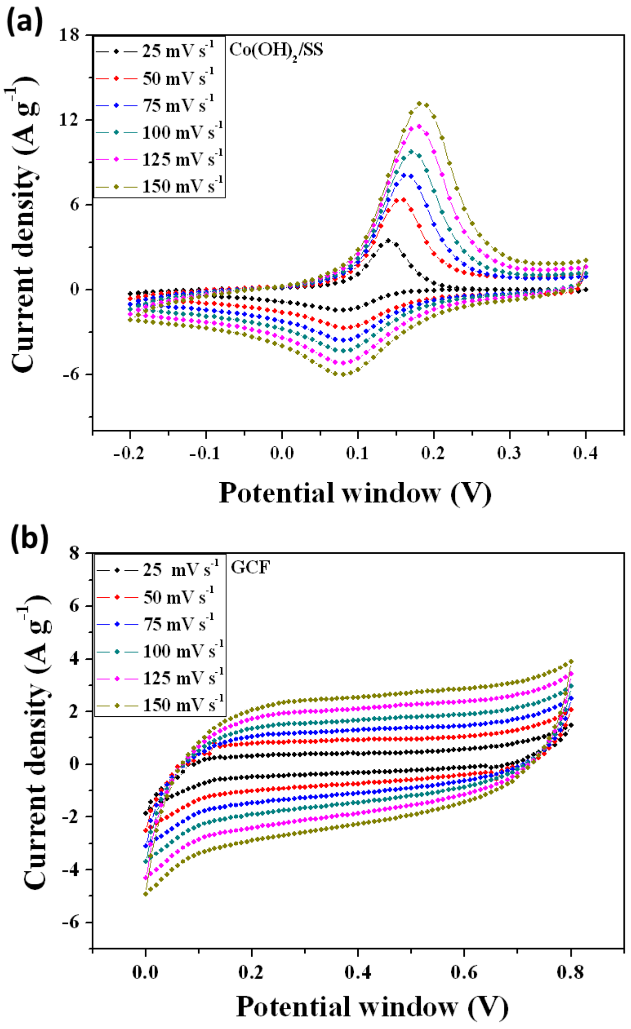

Figure S7. Scan rate dependent CV curves of (a) $Co(OH)_2$/SS electrode within potential -0.2 to 0.4 V and (b) 3D carbon foam within potential window 0 to 0.8 V, in 1M KOH electrolyte. The CV curves of $Co(OH)_2$, on SS electrode, exhibit two intense characteristic redox peaks, anodic peak (+0.15 V) and cathodic peak (+0.04 V) arising from the reversible faradaic reaction in KOH electrolyte. The GCF reveals rectangular shaped CV in 0 to +0.8 V potential window based on typical electrochemical double layer capacitive (EDLC) characteristic.

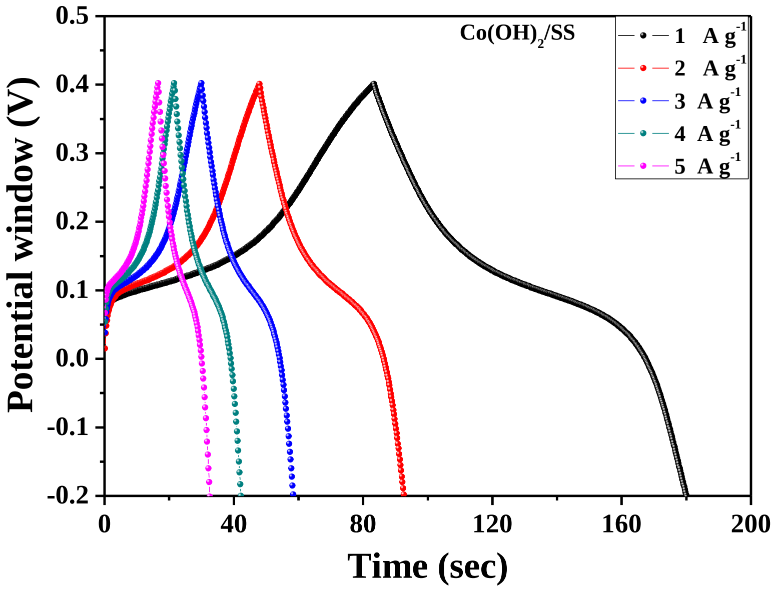

Figure S8 Galvanostatic charge-discharge (GCD) plots of the $Co(OH)_2$/GCF electrode within a potential window of -0.2 to 0.4 V at constant charging current from ~1 to 5 A g^{-1}, reveals non-linear charge-discharge curves originated from pseudocapacitance.
